# Cost accounting in the EDP-supported chemical laboratory

**DOI:** 10.1155/S146392468300022X

**Published:** 1983

**Authors:** H. J. Gibitz

**Affiliations:** Chemical Central Laboratory Landeskrankenanstalten Salzburg A-5020 Austria


Journal of Automatic Chemistry, Volume 5, Number 2 (April–June 1983), pages 79–82

### Cost accounting in the EDP-supported chemical laboratory*

**H. J. Gibitz**

*Chemical Central Laboratory, Landeskrankenanstalten, A-5020 Salzburg, Austria*

### Introduction

In 1978, cost accounting was made compulsory by law for all Austrian public hospitals [1]. Cost accounting at the Chemical Central Laboratory of the Landeskrankenstalten in Salzburg follows the procedures suggested by the law; it is done by the hospital's administration with the help of electronic data-processing (EDP).

The hospital is a teaching hospital with 1600 beds; in addition, there are 700 beds in an affiliated hospital for the mentally ill. The central laboratory carries out all tests requested on blood, urine and other body fluids; the emergency laboratory is part of the central laboratory. Other hospital departments perform haematology, blood group, bacteriology and histology tests. The central laboratory is staffed by three medical doctors, two chemists and 20 technicians.

Three types of cost accounting are performed by the administration: an analysis of kinds of costs; an analysis of cost centres; and an analysis of bearers of costs.

### Analysis of kinds of costs

The analysis of kinds of costs covers all the hospital's costs divided into eight groups. The left-hand side of table 1 shows the result of the 1979 analysis. It can be seen that personnel costs amount to 58% of the total and the costs for the annual rate of depreciation of buildings and equipment amount to some 11·5% of the total costs. (NB. All costs are in 'cost points', which do not refer to any particular currency.)

The central laboratory's costs are presented in the right-hand side of table 1. The laboratory is responsible for 2·91% of the costs of the hospital. Only 38% of the costs of the central laboratory are personnel costs: this is small and a result of mechanization and EDP support.

### Analysis of cost centres

The hospital is divided into 262 cost centres. In the central laboratory there are 10 cost centrès, eight of which are related to functional sections of the laboratory. All direct costs are charged to these cost centres. Indirect costs are collected from two other cost centres and these are distributed amongst the eight main cost centres according to a special ratio. The ratio takes into account the number of tests and the number of technicians at specific cost centres. These indirect costs are called 'inside overhead costs' and they are booked under cost number 14 (table 1). Indirect costs with which the central laboratory is charged by the hospital are labelled 'outside overhead costs', with the cost numbers 11–13 (table 1).

### Analysis of bearers of costs

This is the third type of cost accounting. In the hospital every single patient is a bearer of costs, and in the laboratory each test is a bearer of costs.

As in the central laboratory there are up to 200 different kinds of tests, and it has not been possible so far to put an exact cost account on each test. The costs of each cost centre are uniformly distributed according to the number of tests performed. To do this it is essential that the costs of all tests of one cost centre are similar and also that there is a sufficiently high number of cost centres.

Table 2 shows the central laboratory's eight cost centres, their costs and the numbers of tests in 1979. In the last two columns the costs for one test and one report are calculated. (The differences between tests and reports results from additional tests for standardization, quality control and repeats. The cost of one report includes the cost for the patient's test and an element

*Table 1. Cost accounting in the hospital and in the central laboratory (1979).*

| Kinds of costs | Hospital | | Central laboratory | |
|---|---|---|---|---|
| | Costs $10^3$ | % | Costs $10^3$ | % |
| 01 Personnel | 416701 | 58·0 | 7946 | 38·0 |
| 02 Medical supplies | 94522 | 13·1 | 3598 | 17·2 |
| 03 Non-medical supplies | 33598 | 4·9 | 141 | 0·7 |
| 04 External medical services | 7952 | 1·1 | 1 | 0·1 |
| 05 External non-medical services | 39738 | 5·5 | 739 | 3·5 |
| 06 Power | 7616 | 1·0 | 128 | 0·6 |
| 07 Taxes and fees | 35750 | 4·9 | 48 | 0·2 |
| 08 Annual rate of depreciation (equipment and buildings) | 82856 | 11·5 | 4250 | 20·3 |
| 11 Internal medical services | | | 36 | 0·2 |
| 12 Internal non-medical services | | | 2921 | 14·0 |
| 13 EDP, administration | | | 1086 | 5·2 |
| 14 Inside overhead costs | | | ./. | |
| | 718 836 = 100% → 20894 = 2·91% | | | |

*Table 2. Distribution of costs on eight cost centres of the central laboratory in relation to the number of tests (1979).*

| Cost centre | Total costs $10^3$ | Number of tests | Costs for 1 test | Costs for 1 report |
|---|---|---|---|---|
| 650 Autoanalyser laboratory | 6835 | 575913 | 11·80 | 14·75 |
| 651 Enzyme laboratory | 3405 | 223162 | 15·25 | 19·06 |
| 652 Protein laboratory | 1107 | 19476 | 56·83 | 70·03 |
| 653 Simple manual tests | 1935 | 44745 | 43·24 | 54·05 |
| 654 Endocrinology | 1801 | 22120 | 81·42 | 101·77 |
| 655 Toxicology | 1246 | 1572 | 792·62 | 792·62 |
| 656 Special tests | 1264 | 5844 | 216·29 | 216·29 |
| 657 Emergency laboratory | 3301 | 67226 | 49·10 | 61·37 |
| | 20894 | 960058 | 21·76 | 27·20 |

*This paper is dedicated to Professor Dr Richard Stöhr on his 80th birthday.*

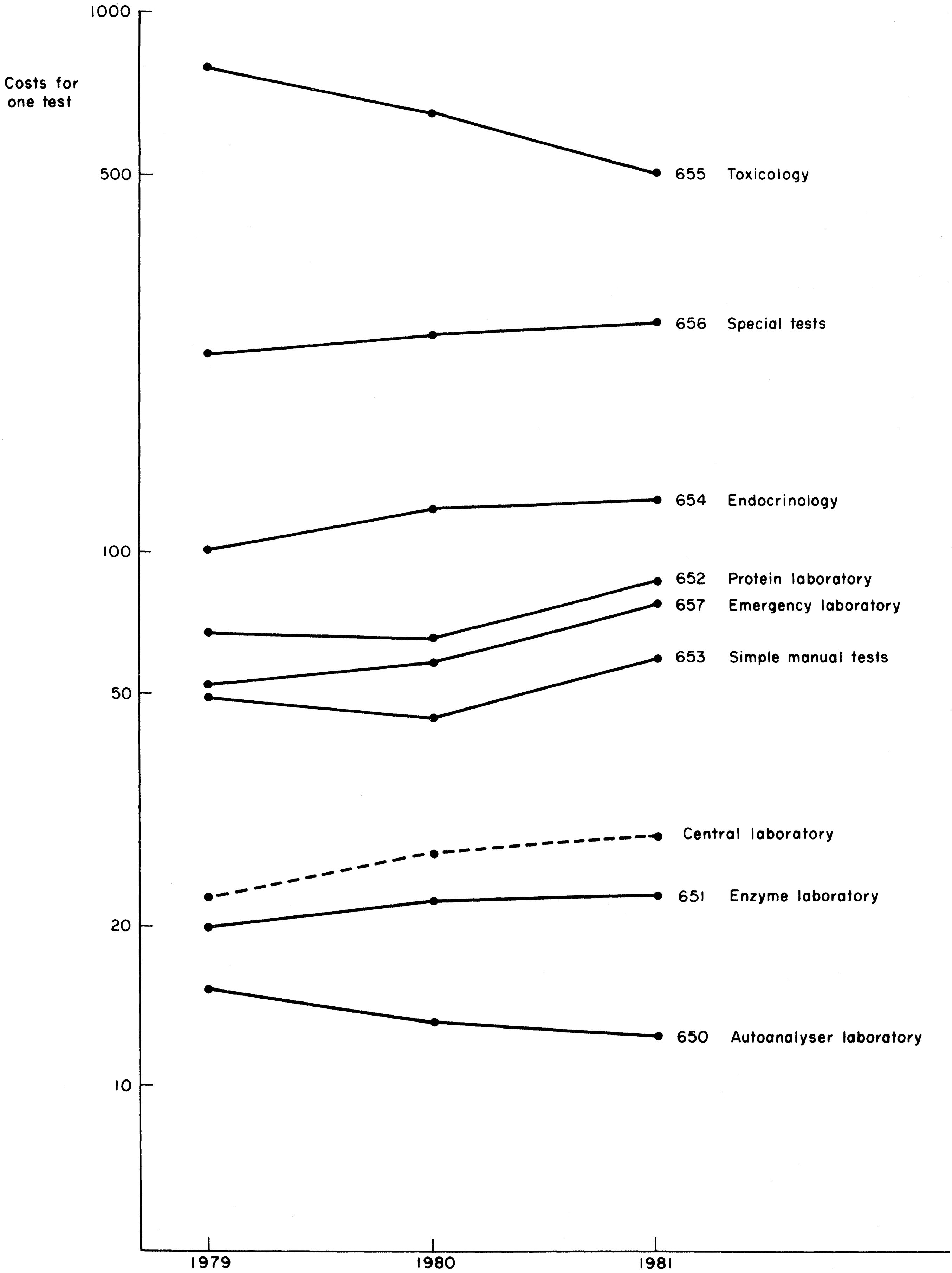

*Figure 1. Evaluation of costs per test in the central laboratory's cost centres (1979–1981.) (The dashed line represents the average costs of all cost centres.)*

of quality-control etc.) These costs differ widely. The lowest costs are found in the autoanalyser laboratory and the highest costs in the toxicology laboratory. In the last row in table 2 the average costs for all tests of all cost centres is given.

### Evaluation of costs of the central laboratory

Figure 1 shows the changes in costs per test over the last three years. The dashed line corresponds to the average cost of all tests of the central laboratory. Their increase from 1979 to 1981 amounts to 10·3%. The total costs of the central laboratory rose by 16·1% during this time, and total costs of the hospital rose by 21·7%. In six of the eight cost centres the cost per test increased but in the autoanalyser laboratory and in the toxicology laboratory there was a significant decrease. The reasons for the decrease are different in both cases.

### *Evaluation of costs in the autoanalyser laboratory*

In the autoanalyser laboratory the decrease in costs is due to the use of on-line data processing of the analogue data from the four autoanalysers linked to an IBM System 7. This effect is most clearly seen in 1980 when the total costs show a marked decrease despite an increase in the number of tests (table 3). As inflation in Austria amounted to 13·1% from 1979 to 1981, a reduction in costs of 24% is a real financial gain. The capital cost of the S-7 computer are included in this cost utility calculation. The decrease of costs is caused by a reduction in staff due to the introduction of on-line data processing. These people (two technicians and a clerk) were freed to work in other parts of the laboratory.

#### *Variable costs in the autoanalyser laboratory*

In a second analysis the variable costs of the different kinds of tests were calculated for 1981 (see table 4). The costs incurred for the technicians were obtained from a study which was carried out according to the Method Time Measurement (MTM) technique [2, 3 and 4]. Details of the technique are given elsewhere [5 and 6]. The measured and calculated working minutes per test are listed in the first column of the table. A so-called 'time of distribution' of 20% is added to account for recreation, short disturbances and the fact that it is impossible to fill short intervals with other work. Then the multiplication with the costs for 1 min of a technician is carried out; the results of this are listed in column 2. The costs for reagents and disposables are given in column 3. The non-variable costs, which are the same for all tests, are listed in column 4. The total costs per test are shown in the last column. The different results in the last column show the error if equal costs are ascribed to all tests performed in the cost centre.

*Table 3. Reduction of costs in the autoanalyser laboratory as a consequence of introducing on-line processing.*

| | 1979 | 1980 | 1981 | 1979–1981 |
|---|---|---|---|---|
| Number of tests | 579547 | 592925 | 613112 | + 5·8% |
| Sum of costs ($10^3$) | 6567 | 6066 | 6188 | − 5·9% |
| Costs per test | 11·80 | 10·23 | 10·09 | −10·9% |
| General rate of inflation | | +6·3% | +6·8% | +13·1% |
| Reduction of costs per test (rate of inflation included) | | | | −24·0% |

*Table 4. Analysis of variable and non-variable costs in the autoanalyser laboratory (1981).*

| | Technicians MTM −min | =costs +20% | Reagents and disposables | Other costs | Total costs |
|---|---|---|---|---|---|
| K | 0·35 | 1·31 | 0·47 | 7·12 | 8·90 |
| Na | 0·35 | 1·31 | 0·47 | 7·12 | 8·90 |
| Cl | 0·35 | 1·31 | 0·94 | 7·12 | 9·37 |
| $HCO_3$ | 0·35 | 1·31 | 1·10 | 7·12 | 9·53 |
| Urea | 0·35 | 1·31 | 0·44 | 7·12 | 8·87 |
| Creatinin | 0·35 | 1·31 | 0·43 | 7·12 | 8·86 |
| Protein | 0·83 | 3·11 | 1·15 | 7·12 | 11·38 |
| Ca | 0·93 | 3·49 | 0·70 | 7·12 | 11·31 |
| P | 0·93 | 3·49 | 0·60 | 7·12 | 11·21 |
| Fe | 0·86 | 3·22 | 1·06 | 7·12 | 11·40 |
| Cu | 0·86 | 3·22 | 0.65 | 7·12 | 10·99 |
| Fe-BC | 1·47 | 5·51 | 1·17 | 7·12 | 13·80 |
| Cholesterin | 0·83 | 3·11 | 2·09 | 7·12 | 12·32 |
| Triglycerides | 0·83 | 3·11 | 1·87 | 7·12 | 12·10 |
| Bilirubin | 1·35 | 5·06 | 0·87 | 7·12 | 13·05 |
| Uric acid | 1·35 | 5·06 | 3·38 | 7·12 | 15·56 |
| | | | | | 10·10 |

### *Evaluation of costs in the toxicology laboratory*

A decrease in cost per test is also apparent in the toxicology laboratory (see table 5). In this case the cause is the different kind and number of tests performed. The number of tests for therapeutic drug monitoring, other enzyme immunoassays (EIA) and infrequent chemical tests increased from 1979 to 1981, while the number of toxicological drug screenings remained nearly the same. The 35% decrease on costs for all tests results from an unequal distribution of the costs on the four groups of tests. The exact analysis of cost shows (table 6) that the variable costs for personnel, and consequently the total costs for the toxicological drug screening, are 40% higher than the costs in the other three groups, whose total costs are nearly equal. The problem can only be solved by introducing an additional cost centre for toxicological drug screening.

In the column 'other costs', the outside overhead costs are included. Up to now they were distributed equally on all central laboratory cost centres. In the toxicology laboratory they are extremely high per test because the number of tests is very small. In future, the outside overhead costs will be distributed according to the ratio used for inside overhead costs. Consequently there will be a considerable decrease in the 'other costs' per test in the toxicology laboratory and a small increase in other costs for the other cost centres, which carry out more tests.

### Distribution of the central laboratory's costs to the hospital's clinical departments

The task of the cost accounting in the hospital is to correctly allocate to patients all costs incurred during their stay. This problem is not yet fully solved in our hospital. The costs of the cost centres of the central laboratory must only be charged to the cost centres who requested the tests.

Table 7 shows a survey of the distribution of the costs of the central laboratory to the various clinical departments. The costs per bed and day differ widely—this is due not only to the various medical tasks but also to local situations. The costs per test reflect the variety of tests available, although the range on offer is not excessively large. There is only one exception: the paediatric department requests only expensive emergency tests and microlitre tests.

*Table 5. Number of tests and costs in the toxicology laboratory (1979–1981).*

| Kinds of tests | 1979 | 1980 | 1981 | 1979–1981 |
|---|---|---|---|---|
| Toxicology: drug screening | 453 | 359 | 452 | − 0·3% |
| Therapeutic drug monitoring | 466 | 652 | 792 | + 69·9% |
| Other EIA (CEA, Ferritin) | 578 | 1267 | 2476 | +328·4% |
| Clinical chemistry | 75 | 111 | 145 | + 93·3% |
| Total number | 1572 | 2389 | 3869 | +147·3% |
| Total costs | 1246000 | 1568000 | 1977000 | + 58·7% |
| Cost per test | 793 | 656 | 511 | − 35·6% |

*Table 6. Distribution of costs in the toxicology laboratory (1981).*

| Costs per rest | Technicians | Reagents and disposables | Other costs | Total costs |
|---|---|---|---|---|
| Toxicology: drug screening | 315 | 138 | 322 | 802 |
| Therapeutic: drug monitroing | 34 | 151 | 322 | 519 |
| Other EIA (CEA, Ferritin) | 19 | 117 | 322 | 458 |
| Clinical chemistry | 75 | 138 | 322 | 535 |
| | | | | 511 |

### Conclusions

The cost-accounting system presented in this paper is relatively simple. One of its merits is that it has already worked for three years, and the results of three years can now be compared.

The calculations are sufficiently exact to pass the costs of the laboratory tests on to the patients or on to their respective cost centres. Within the central laboratory, cost accounting gives a good survey of the distribution of costs to the laboratory's functional units. It is an important tool for laboratory management and a great help in official discussions about additional personnel, new equipment and accommodation needs.

The results show a very favourable relation of costs to monetary utility in the central laboratory, in comparison with the other hospital departments. Cost accounting is an important precondition for future investigations about the non-monetary utility of laboratory tests.

*Table 7. Annual distribution of laboratory costs (clinical chemistry) to the hospital's departments (1981).*

| Clinical departments | Beds | Costs ($10^3$) | Costs per bed and day | Cost of one test |
|---|---|---|---|---|
| (1) Surgery | 268 | 2902 | 30 | 23 |
| (2) Vascular surgery | 36 | 634 | 48 | 26 |
| (3) Facial surgery | 25 | 329 | 36 | 33 |
| (4) Orthopaedics Ophthalmology Laryngology | 163 | 771 | 13 | 24 |
| (5) Gynaecology Obstetrics | 231 | 640 | 8 | 23 |
| (6) Urology | 76 | 1247 | 45 | 27 |
| (7) Internal medicine | 289 | 5518 | 52 | 27 |
| (8) Paediatry | 254 | 1156 | 13 | 76 |
| (9) Dermatology | 79 | 594 | 21 | 23 |
| (10) Neurology Psychiatry | 729 | 2637 | 10 | 23 |
| (11) Pulmology Radiotherapy | 74 | 554 | 20 | 30 |
| (12) Intensive care unit | 4 | 276 | 189 | 29 |

### References

1. Krankenanstaltenkostenrechnungsverordnung, *Bundesgesetzblatt für die Republik Österreich*, **328** (1977).

2. Maynard, H. B., Stege-Merten, G. J. and Schwab, J. L., *Methods-Time Measurement* (McGraw-Hill, New York, 1948).

3. Covert, R. P., *The Journal of Methods-Time Measurement*, **4** (1976), 23.

4. Gaubl, K., *REFA-Nachrichten*, **30** (1977), 1.

5. Gibitz, H. J., *Berichte der Österr. Gesellschaft für klinische Chemie*, **3** (1980), 44.

6. Gibitz, H. J., In preparation.

### MEETING

*Computing in Clinical Laboratories*

This is the fourth international meeting on Computing in Clinical Laboratories and will be held in Breda, The Netherlands, from 24–26 August 1983. The programme is designed for clinical laboratory workers and computer scientists and is divided into three main sessions: Introduction of computer systems into pathology laboratories; Computer networks; and Computer-assisted interpretation of laboratory data.

*Further details from Mr R. C. J. Galle, Stichting Medische Laboratoria, Bergschot 69, 4817 PA Breda, The Netherlands.*

